# 'No sister, the breast alone is not enough for my baby' a qualitative assessment of potentials and barriers in the promotion of exclusive breastfeeding in southern Zambia

**DOI:** 10.1186/1746-4358-3-26

**Published:** 2008-11-05

**Authors:** Eli Fjeld, Seter Siziya, Mary Katepa-Bwalya, Chipepo Kankasa, Karen Marie Moland, Thorkild Tylleskär, the PROMISE-EBF Study Group

**Affiliations:** 1 Centre for International Health, University of Bergen, Norway; 2 Department of Community Medicine, University of Zambia (UNZA/SOM), PO Box, 50110, Lusaka, Zambia; 3 Department of Paediatrics and Child Health, University of Zambia (UNZA/SOM), PO Box, 50110 Lusaka, Zambia; 4 Faculty of Health and Social Sciences, Bergen University College, Norway; 5 see complete list at end of article

## Abstract

**Background:**

Appropriate feeding practices are of fundamental importance for the survival, growth, development and health of infants and young children. The aim of the present study was to collect baseline information on current infant and young child feeding practices, attitudes and knowledge in Mazabuka, Zambia, using a qualitative approach.

**Methods:**

The study was conducted in Mazabuka, 130 km south of Lusaka in Zambia in January and February in 2005. Nine focus group discussions with mothers and a total of 18 in-depth interviews with fathers, grandmothers, health staff and traditional birth attendants were performed in both rural and urban areas.

**Results:**

Breastfeeding was reported to be universal, the use of pre-lacteal feeds appeared to be low, colostrum was rarely discarded, and attitudes to and knowledge about exclusive breastfeeding were generally good. However, few practised exclusive breastfeeding. The barriers revealed were: (1) the perception of insufficient milk, (2) the fear of dying or becoming too sick to be able to breastfeed, (3) convention, (4) the perception of 'bad milk' and (5) lack of knowledge on the subject. The health staff and traditional birth attendants were the most important actors in transmitting knowledge about infant feeding to the mothers. Both categories appeared to have updated knowledge on child health and were well respected in the society. Fathers and grandmothers tended to be less knowledgeable on novel subjects such as exclusive breastfeeding and often showed a negative attitude towards it. At the same time they had considerable authority over mothers and children and infant feeding decisions. The rural population was in general less educated and more prone to conventional non-exclusive feeding practices.

**Conclusion:**

The message that exclusive breastfeeding (EBF) is beneficial for child health had reached the health workers and was taught to mothers. However, conventions and expectations from family members in this Zambian community were important barriers in preventing the message of EBF from being translated into practice. The deep-rooted beliefs that prohibit EBF need to be addressed in projects and campaigns promoting EBF.

## Background

Each year more than 10 million children under the age of five years die, mainly from one of a short list of causes, and the majority live in low-income countries [1]. Millennium Development Goal number 4 is to reduce child mortality by two thirds by 2015 [2]. In Sub-Saharan Africa, where almost half of all deaths in children aged less than five occur, the decrease in mortality rates has slowed down, and in some countries the mortality rate has even increased. The causes for this change in child survival are many and include rising poverty, fragile health systems, HIV/AIDS and malnutrition. Malnutrition is estimated to be the underlying cause of 54% of under five mortality [3]. Appropriate feeding practices are of fundamental importance for the survival, growth, development, health and nutrition of infants and young children. It is argued that promotion of exclusive breastfeeding (EBF) is the most effective child health intervention currently feasible for implementation at population level in low-income countries [4]. EBF could lower infant mortality by 13%, and by an additional 2% were it not for the fact that breastfeeding may transmit HIV [4]. For many HIV-positive mothers, the risk of transmitting HIV to their children through breastfeeding outweighs the risk of the infant dying from other infectious diseases if not breastfed [5,6]. Despite the almost universal practice of breastfeeding, most women do not practise EBF for the recommended six months [7]. Research shows however that good infant feeding counselling and support, provided by health care staff or peers, can improve the rates of EBF [8-11].

The aim of the present study was to collect baseline information about current knowledge, attitudes and practises related to the feeding of infants and young children, with a special focus on EBF. This information was intended to assist in designing an intervention to improve these practices.

## Methods

This study was conducted in January and February 2005 and included a total of nine focus group discussions with mothers and a total of 18 in-depth interviews with fathers, grandmothers, health staff and traditional birth attendants, conducted in both rural and urban areas of Mazabuka District, Zambia.

### Study area

Mazabuka District is situated 130 km south of Lusaka, the capital of Zambia, on the main road to Livingstone. Mazabuka is one of 11 districts in Southern Province and has a population of approximately 200 000 [12]. The urban participants in this study were all residents of the Kaonga community, situated in central Mazabuka. The community comprises low and middle class inhabitants according to Zambian standards. The rural participants came from villages covered by the Kaonga clinic catchment area. Compared to the urban population, the rural population was less educated, a larger proportion was illiterate and they tended to have more children. The main languages spoken in the area are Tonga, Bemba and Nyanja.

### Focus group discussions (FGDs)

Focus groups discussions actively make use of group interaction. Participants are able to build on each other's ideas and comments, thus the discussions give insight into collective meanings attached to a specific phenomenon that cannot be elicited by questioning individuals. It's purpose is to reveal both consensus and diversity in the participants' knowledge, experiences, preferences and assumptions [13]. As the aim of this study was to collect baseline information about knowledge, attitudes and practices relevant to infant feeding, FGD was chosen as the main data collection tool.

Nine FGDs were conducted; four in the urban area and one in each of the five rural villages included in the study. In order to make the groups as homogenous as possible and to reflect differences in the experiences and perceptions between different age groups, we conducted five FGDs with women under 25 years of age, and four with women aged 25 years and above.

In the urban area, the Kaonga community, the first author and one of the research assistants went from door to door in order to recruit mothers to the study. In the rural areas the research team joined the mobile clinic from Kaonga Health Centre when they visited the different villages. The mothers were already waiting for the nurses when we arrived at each village, and the nurses asked 8–10 of them if they were willing to participate in the study. The inclusion criteria for the FGDs were: mother of children younger than two years of age, or older children if she was still breastfeeding. A few of the women in the rural area chose not to participate because they feared their husbands' disapproval. Verbal informed consent was obtained after the participants had been informed about the study objectives.

The FGDs, with 6–10 participants in each group, were conducted outdoors or in a local guest house, school house or church. After each session the women received some refreshments and a small travel reimbursement. The focus groups were facilitated by a social scientist, who was trained and experienced in conducting FGDs and was fluent in three local languages (Tonga, Bemba, Nyanja) and English. All four languages were used interchangeably in the discussions. Prior to each group discussion the facilitator discussed the issue of confidentiality with the participants; she also made sure that there was a relaxed atmosphere before the discussion started.

A topic guide with a written list of questions and probes was used throughout all the FGDs and the moderator was well informed of all areas of interest. Topics covered were initiation of breastfeeding, practices regarding colostrum, use of pre-lacteal feeds, complementary food, cessation of breastfeeding, exclusive breastfeeding, no breastfeeding, breastfeeding problems, insufficient milk and influential persons. The sessions were tape-recorded. A scribe took detailed notes to help with transcription. The first author supervised all the discussions and acted merely as an observer. Each session lasted for 1.5–2 hours. At the end of each FGD, there was a debriefing between moderator, scribe and the first author to discuss the most important themes and possible differences from previous FGDs. After nine FGDs had been conducted we felt that no new information was emerging and that saturation had been reached.

### In-depth interviews

In-depth interviews are used to explore the meanings of social phenomena as experienced by individuals, in their natural context [14,15]. During the study it became apparent that in addition to FGDs with mothers, there was a need to map the viewpoints and influence of people surrounding the breastfeeding mother. In all, 18 in-depth interviews were conducted; six with fathers, four with grandmothers, four with traditional birth attendants (TBAs) and four with nurses. The potential participant was approached by the first author and the interviewer, and asked if he or she was willing to participate in the study. The inclusion criteria were, being a father or a grandmother of a child less then two years or still being breastfed, being an active TBA in health education and birth care or a nurse working at an 'Under five clinic'. In each category of informants there were representatives from both the urban and rural populations and from different age groups. A semi-structured interview design was used, and an interview guide was prepared for each key informant category. The guides covered the same topics as the FGDs, but the questions were designed to explore the individual perceptions and experiences of the participants.

The interviews were conducted by five investigators. The majority of the interviews conducted in a local language were done by the facilitator of the FGDs, but on three occasions another research assistant conducted the interview. If the key informant was fluent in English, the interview was conducted by the first author, which was the case in six of the interviews. The interviews were tape-recorded and ranged in length from 45 minutes to two hours.

### Data analysis

The FGDs and the in-depth interviews were transcribed verbatim and translated into English by the facilitator of the FGD groups and then carefully read through by the first author. After all the interviews were completed, they were read through several times by the first author, who also did the data analysis [16,17]. Most information categories were generated deductively from the question guide. The information in each FGD was summarised and grouped according to these predefined information categories. Illustrative quotations were selected. During this process, new categories, e.g. barriers to EBF, emerged and were added to the analysis. Towards the end of the data analysis, the urban and rural FGDs were grouped into an urban and a rural cluster and similarities and differences within and between the clusters were investigated.

The in-depth interviews were analyzed simultaneously with the FGDs, applying the same strategy of analysis. The information categories obtained in the FGD analysis were used to organize the information in each interview. The interviews were grouped together according to the informant category: fathers, grandmothers, TBAs and nurses. Finally, the information obtained in the in-depth interviews was merged with the analyzed FGDs, so that each information category contained information collected through both FGDs and in-depth interviews.

All original data were re-assessed after analysis in order to detect any concepts or information that had been missed.

Within each information category, the findings will be presented in the following order when information from the particular group applies: (1) FGDs with urban mothers, (2) FGDs with rural mothers, (3) in-depth interviews with grandmothers, (4) in-depth interviews with fathers, (5) in-depth interviews with nurses and TBAs.

### Ethics

Ethical approval for the study was obtained from the Regional Committee for Medical Research Ethics, West Norway (REK Vest) and the University of Zambia Research Ethics Committee. Permission to conduct the study was sought from the Central Board of Health and the District Health Management Team in Mazabuka (Zambia).

## Results

Table 1 gives an overview of the different groups interviewed and their view in brief on the topics discussed.

**Table 1:** Overview of the different groups interviewed and their views in brief on the topics discussed

	Urban mothers (4 focus groups)	Rural mothers (5 focus groups)	Grandmothers (4 in-depth interviews)	Fathers (6 in-depth interviews)	Health staff & TBA (4 in-depth interviews of each)
Colostrum and early initiation of breastfeeding	-Start breastfeeding immediately -Colostrum: -Gave it -Protection -Nutritious -Dirty	-Start breastfeeding immediately -Colostrum: -Some expressed it -Dirty -Protection	-Start breastfeeding immediately -Should give colostrum	-Start breastfeeding immediately	-Start breastfeeding immediately -Taught to give colostrum
Pre-lacteals	-Negative	-Negative -A few practiced it -Gave water			-Discouraged it
Exclusive breastfeeding	-Positive -Knowledgeable -Practiced by few -Breast milk insufficiency main barrier	-Positive -Knowledgeable -Practiced by few -Fear of dying main barrier	- Skeptical - Breast milk insufficiency and fear of the mother dying	-Not well informed -Skeptical -Child needed extra food	-Part of the education program -Few mothers practiced it
Water and complementary foods	-Mealie meal -Water -Commercial porridge -Formula milk	-Mealie meal -Water	-Negative	-Mealie meal -Water -Commercial porridge -Formula milk	-Emphasized not to give before 6 months
Breastfeeding problems	-Sores -Not sucking -Engorgement -Breast milk insufficiency -Child not satisfied -"Bad milk" -Lead to giving complementary foods -A barrier to EBF	-Sores -Not sucking -Engorgement -Breast milk insufficiency -Child not satisfied -"Bad milk" -Lead to giving complementary foods -A barrier to EBF	-Sores -Engorgement -Child not satisfied -Need for complementary foods	-Breast milk insufficiency -Child not satisfied -Need for complementary foods	-A barrier to EBF
Cessation of breastfeeding	-At 18–24 months -Keep the child satisfied -Bring the child to relatives	-At 18–24 months -Bring the child to relatives -Traditional methods			-Gradual -Not separate from mother -Nutritious food
Reasons to stop breastfeeding earlier than 18–24 months	-Mother very sick (HIV/AIDS) or infectious -"Bad milk" -Sore nipples -New pregnancy	-"Bad milk" -New pregnancy	-Mother very sick (HIV/AIDS) -New pregnancy		
Not breastfeeding at all	-Negative to -Sick mother -Lack of milk -Sores on breasts	-Negative to -Sick mother -Lack of milk -Sores on breasts	-Not acceptable	-Not acceptable	
Infant feeding advice and support	-Clinic -Faith in nurses -People experienced with children	-Older people		-Provided material support	-Father -Mother-in-law

### Colostrum and early initiation of breastfeeding

There was a general opinion among all the participants, in both the focus groups and the in-depth interviews, that all mothers should breastfeed and that they should start immediately after birth. Both the nurses and the TBAs taught and encouraged the mothers to initiate breastfeeding instantly after giving birth.

Among the urban mothers, colostrum was generally perceived as good, protective for the child and nutritious.

"The first milk is very good, because it protects the baby against diseases. A baby can not contract so many diseases when it is breastfeeding. So like in hospitals, they advise us to breastfeed our babies just after delivery. It's because they don't want the baby to miss that fluid." (Urban FGD # 1, respondent # 5)

None of the urban mothers reported having expressed and discarded the colostrum. A small number of the urban women said that colostrum was not good; arguing that it might be dirty or that it is just water.

In the rural focus groups, a few of the mothers reported having expressed the colostrum and discarded it; they held that the first milk was dirty and they feared that it might make the child sick. The practice of expressing and discarding milk was also mentioned.

"It differs because that is just water, and please, we should pay attention to our breasts when we are pregnant, there is that dirt on our breasts. So when we deliver, that fluid should be squeezed out so that it completely comes out, because if it doesn't, the child might get sick." (Rural FGD # 5, respondent # 2.)

However, most of the rural mothers claimed that they gave the colostrum, and some emphasized its protective effects. All the grandmothers spoke in favour of the colostrum and said it should be given.

### Pre-lacteal feeds

The majority of the mothers in the focus groups were against giving anything to the baby before initiation of breastfeeding. Urban mothers claimed that they did not give any pre-lacteal feeds. Some of the rural mothers reported that they gave it; some just gave water to wet the mouth of the newborn, others added herbs or mixed pounded groundnuts to the water. The rationale for giving pre-lacteal feeds was that there was no milk in the mother's breast.

"Yes, I normally give water. It's . . . it's to make the throat wet. Not for the throat to be dry, because milk at that time has not yet started coming out." (Rural FGD # 5, respondent # 4)

### Exclusive breastfeeding

All the mothers in this study were familiar with the concept of EBF. In general they spoke in favour of it. They agreed that a child could survive on breast milk alone for the first six months. Nevertheless, EBF was practised only by a few. When asked why they should exclusively breastfeed for six months, the mothers mentioned that food would cause diarrhoea.

"Food give diarrhoea, and again, the intestine is still small. So, if you introduce porridge, the baby's stomach pains. So that is why they don't advise us to give other foods apart from milk for six months." (Urban FGD # 1, respondent # 6)

In the urban area, the main barrier to EBF that came up in the discussions was the perception of the child not being satisfied.

"As the baby grows you find that they will start crying of hunger most of the times because they are not getting satisfied on milk, because they are now grown big. So, you find that even if they tell you at the clinic to practise exclusive breastfeeding, when you reach home you give them other food supplements, like orange juice or milk." (Urban FGD # 2, respondent # 1)

The rural mothers also expressed the fear that the milk was not enough to satisfy the child, but here an additional obstacle to EBF was raised; the fear of the mother becoming sick and dying, leaving a child who had not become accustomed to taking anything except breast milk.

"No, it was enough (milk), I just thought that I could get sick and so I wanted my child to get used to eating other foods, so that even if I die he will be able to survive." (Rural FGD # 2, respondent # 8)

This fear about insufficient milk was also a major concern for the grandmothers. The majority of the grandmothers responded negatively to EBF, Some of them used the argument that the child needed to get used to eating foods in case the mother fell sick and maybe died. They would all recommend their daughter/daughter-in-law to start giving the child other foods before six months.

"I would tell them to give other foods, but when I tell them, they say that's not what they are told at the clinic; at the clinic they refuse them to give babies other foods." (Grandmother # 2)

"How can a baby be healthy with breast milk only? They need some porridge as well and a bit of water too." (Grandmother # 1)

Some of the fathers had not heard about EBF. Among those who were informed, most were not in favour of it; they said that the mother might not have enough milk, and that the child needs other food. Some said it was acceptable as long as the mother had sufficient milk, others would not allow their wives to practise EBF.

"No, because the baby needs other foods. Because from ...let me say 2–3 months, it means that the baby is able to eat . . . only milk; within just a few minutes he will feel hungry, so within this time it's good to give porridge with milk." (Father # 1)

EBF was part of the education programme at the clinic. The nurses had updated knowledge of the subject. Advantages mentioned were that the child would grow well and be healthy, and the mother would be assisted with family planning. There was no mention of the risk of HIV transmission and the protective effect of EBF as compared to mixed feeding. According to the nurses, EBF was practised by some, but they also said that many would report to the health personnel that they followed EBF, but in reality they gave water and started with porridge before six months.

"Okay, some of them they are not open. It's like, when you are teaching them, it's like they will do what you are telling them. But maybe, when a child comes back, he is not well, you ask them: 'What did you give this child?' That is when they will tell you: 'No sister, the breast alone is not enough for the baby; the baby needs some other food stuffs as well."' (Nurse # 2)

According to the nurses, the main barrier to EBF was the perception that breast milk was not satisfying the child.

"They still have that fear that they can get pregnant, the baby is thirsty, the milk is not enough for the baby, and at least they need something heavy to feed their baby that will last a long time." (Nurse # 3)

The nurses identified a potential conflict between their advice as health personnel and the opinions of close relatives. These differences often put the mother in a difficult situation.

"The clients themselves would rather prefer to follow the health workers' advice than that of the relative. But it is only that they wouldn't like to disappoint their relatives as well." (Nurse # 3)

The TBAs had updated knowledge on EBF and expressed a very positive attitude towards it.

"Yes, it's very good (EBF). Because a baby who breastfeeds for six months grows well. But if you introduce other foods, he develops stomach pains. Now that's when the diseases are contracted and the baby might develop diarrhoea. So that's why we advise mothers to breastfeed their children because in breast milk all the medicine and all foods are there. So, the baby should be breastfed for six months, without being given other foods." (TBA # 1)

In this area of Zambia, breastfeeding support groups had been established at some clinics. The members of the groups encouraged each other to breastfeed exclusively. The mothers held meetings and supported and educated their peers about EBF. Mothers who had succeeded with EBF acted as role models for the others. The health personnel made use of these groups when teaching about EBF.

### Water and complementary foods

The main complementary food given mentioned by all the interviewees was light *mealie meal *(maize flour) porridge which was introduced at the age of 2–6 months. It was common practice to enrich it with oil, sugar fortified with vitamin A, salt, pounded groundnuts or/and pounded *kapenta *(small dried fish), increasing the nutritional value markedly; however, the additives were mainly chosen on the basis of affordability and accessibility. Other complementary foods mentioned were egg yolk, cows' milk, bean soup, and fruits such as oranges, lemons and bananas. Another item mentioned by many was plain water, which was typically given before introducing the first complementary foods. When the child became older, six months or more, *nshima *(thick maize flour porridge) was usually given. *Cerelac *(commercial porridge) was mentioned by several of the urban mothers. If they could afford it, they would give it to their child, usually as the first complementary food the child received. The urban mothers also seemed to be very positive towards formula milk; if they could afford it, most of them would give it. The same idea seemed to prevail among the rural mothers, but *Cerelac *and infant formula were less frequently mentioned.

The grandmothers did not seem to be in favour of introducing complementary food early, saying the child was too small to eat; it was only accepted if there was insufficient milk. The fathers, on the other hand, seemed positive towards complementary foods, mentioning porridge, but also infant formula and *Cerelac*.

Both the nurses and the TBAs emphasized that the child should not be given any complementary foods or water before six months.

### Breastfeeding problems

The breastfeeding problems mentioned by the mothers in the FGDs can be divided into three main categories: (1) general breastfeeding problems such as technique, infections and attitudes; (2) breast milk insufficiency; and (3) 'bad milk'.

"(. . .) for some, after delivery, they normally develop sores on their breasts and then for some, the milk does not completely come out. It's just dry. So in the end that becomes a problem." (Urban FGD # 3, respondent # 3)

The mothers in the FGDs mentioned solutions such as: stop breastfeeding and start feeding the child with boiled cow's milk, seek help from the hospital, or (as mentioned by some rural mothers) visit a traditional doctor to be tattooed on the breasts to make the milk start flowing. Several of the rural mothers said they would seek advice from their own mother, others from their grandmother or older people in general.

Breast milk insufficiency was another perceived problem, apparently experienced by most of the mothers. When they did not think that their child was being satisfied by breast milk alone, they would start giving other feeds.

All the grandmothers were familiar with the problem of breast milk insufficiency and many could tell about a daughter who had experienced it.

In addition to the general concern about milk insufficiency, many mothers in the FGDs reported that breast milk can turn 'bad'. It could turn 'bad' for several reasons: if the mother was sick, especially from HIV, the milk was regarded as 'bad'.

"In situations of HIV/AIDS, they (the health personnel) advise not to breastfeed when the baby is born. They don't tell you not to breastfeed exactly, it is up to you to decide whether you breastfeed, otherwise the disease will go to the baby." (Urban FGD # 2, respondent # 2)

Several said that breast milk would become 'bad' if a mother stopped breastfeeding for a while; it would turn sour and be harmful for the child. If the child got sick, many of the mothers regarded it as a sign of the milk being 'bad'; the milk had made the child sick. In the rural area the most common reason mentioned was that the milk would become 'bad' after conception.

"Yes, when you fall pregnant it gets dirty." (Rural FGD # 1, respondent # 5)

'Bad milk' was considered dangerous for the child; it might get sick and die. There was disagreement as to whether or not 'bad milk' should be given. Some said it was acceptable just to squeeze out the 'bad' milk and then continue breastfeeding. Others said it should not be given, while still others said there was nothing they could do; the child needed the breast milk. Often it was not the mother herself who wanted to stop breastfeeding; it was decided by a close relative, the father of the child or the nurse.

### Cessation of breastfeeding

It was stated that children generally stopped breastfeeding between 18 and 24 months of age, and at this point cessation was often reported to be abrupt. Most of the mothers focused on giving the child a lot of foods and buying drinks, making sure it did not grow hungry. A non-alcoholic local brew (*Munkoyo/Chibwantu*) made of mealie meal and roots was normally given, as well as scones and biscuits. Many stressed the importance of giving the child warm food. Separation of the mother and infant was reported to be an effective way of achieving breastfeeding cessation. The child would normally be brought to relatives; usually the mother's parents, and would be left there for about a month.

"When a child wants to stop, you take him to someone's place; like at his grandparents place, or if I have an elder sister or brother, I can take him there." (Urban FGD # 3, respondent # 6)

This practice was discussed in the FGDs and it appeared to be common in the rural setting. There were also some conventional weaning methods not mentioned by the urban mothers, such as putting chilli on the breasts to discourage the child from suckling, or saying there is an insect on the breast, scaring the child away from breastfeeding.

Few of the nurses and TBAs agreed with the breastfeeding cessation methods evoked in the FGDs. They said that weaning should be done gradually and they disapproved of the practice of leaving the child with relatives. In contrast to the biscuits, scones and traditional drinks mentioned by the mothers as weaning foods, the nurses emphasised the importance of giving the child nutritious and affordable food when breastfeeding was stopped, such as groundnuts, *kapenta *and beans.

### Reasons for stopping breastfeeding earlier than 18–24 months of age

In general, the urban and rural mothers agreed that if the mother was very sick, the majority mentioning HIV/AIDS, or if the disease could be contracted by the child, she should stop breastfeeding.

"(. . .) but after delivery, she was tested for HIV, and she was found to be positive. So, she was advised not to breastfeed the baby. So that baby grew up on other formulas." (Urban FGD # 1, respondent # 6)

Other reasons mentioned were sore nipples, the suspicion of the milk being bad or a new pregnancy. Some also mentioned embarrassment.

"To others it's pride. They would not want to show their breasts in public. So she will introduce him to bottle feeding." (Rural FGD # 1, respondent # 1)

Opinions were divided on the advisability of continuing breastfeeding when the mother was pregnant. Many mothers argued that a new pregnancy would turn the milk 'poisonous' and hence it would be dangerous for the breastfeeding child. On the other hand, one mother said that you cannot stop breastfeeding; the child would die.

In the rural area it appeared to be common practice to stop breastfeeding when the mother became pregnant. The grandmothers concurred with the rural mothers: a mother with AIDS should not continue to breastfeed, neither should a woman who is pregnant.

### Not breastfeeding at all

The most common reason mentioned for not breastfeeding was the mother being sick. Other stated causes were sores on the breasts, pain while breastfeeding or lack of milk at birth.

A few of the mothers in the FGDs mentioned pride, laziness or being too busy to breastfeed. In general, the mothers expressed negative attitudes about not breastfeeding, especially if the mother was healthy.

"It's bad because a child is supposed to be breastfed." (Urban FGD # 3, respondent # 6)

One mother was positive about not breastfeeding, arguing that white European people bottle feed.

"It's good because I hear whites normally use a feeding bottle so that he (the child) grows well. And they get used to their father more than their mother. It's good." (Urban FGD # 3, respondent # 5)

Generally, the grandmothers would not accept their daughter or daughter-in-law not breastfeeding and would demand an explanation from her.

"I would ask her why she is not breastfeeding. I can also tell her to start breastfeeding, whether sick or not, I can tell her to start breastfeeding." (Grandmother # 1)

Almost every father reacted with disapproval of a mother who did not breastfeed her child.

"They don't do a good thing. If milk is there in their breasts and they just don't want to breastfeed, then they are not doing a good thing." (Father # 1)

### Infant feeding advice and support

The women in the FGDs in general stated that their source of support and advice on feeding was the health workers at the clinic. The mothers expressed great confidence in the nurses; they were educated and were considered knowledgeable.

"You can only get the best advice from trained health workers, not anyone else. Sometimes our parents can give us wrong advice, because they are not trained. But if we go to those that are trained, like doctors or nurses; they will give us the best advice." (Urban FGD # 1, respondent # 7)

The mothers also mentioned their parents, friends with small children, neighbours, people experienced with children and their grandmother as advisors on infant feeding matters. The father of the child was not mentioned as a person from whom to seek advice, but was cited by many as a provider of material support. This was also reflected in the in-depth interviews with the fathers.

Women in the rural FGDs tended to get support from close relatives rather than from nurses. Living in a rural area they commonly had more limited access to a health unit, but lived close to their own parents and/or their parents-in-law.

"When you have the first child, your mother teaches you what to do." (Rural FGD # 5, respondent # 7)

When the nurses were asked who had influence on the mother, their spontaneous answer was the father of the child and the mother-in-law. Fear of mothers-in-law possibly pursuing old and infelicitous traditions was also expressed.

"Sometimes it is even better you involve the husband as well in the health education, the one who gets paid you know. Because sometimes the husband is well paid, but maybe he drinks a lot, he gives very little to the mother, the wife, so there is no way that the wife can afford. He needs to understand that it is important for those children at least to get nutritious kind of food, so that they grow well." (Nurse # 2)

## Discussion

In this study we collected qualitative data on infant and young child feeding, with a special focus on EBF. The aim was to use the information gathered to assist in designing an intervention to improve infant and young child feeding practices. In which ways can EBF best be promoted in this area? In the interactions, the mothers brought up two major obstacles to exclusive breastfeeding: (1) the perception of insufficient milk and (2) the fear of dying or becoming too sick to be able to breastfeed. Three further obstacles were revealed: (3) the convention of mixed feeding, (4) the perception of 'bad milk' and (5) lack of knowledge on the subject, in particular among the grandmothers and fathers.

The information collected in this study can be used as a basis for suggesting improvements to health education in Mazabuka to promote EBF better (Table 2).

**Table 2:** Suggested improvements in the content of the health education and ways to implement the recommendations

**Suggested improvements in the content of health education**
• Explain the underlying physiology, rationale and principles of exclusive breastfeeding including:
◦ No need for water
◦ Breast milk production increases by frequent suckling and decreases with less frequent suckling
◦ 'Crying baby' is not necessarily a sign of insufficient feeding, not necessary to give complementary food
◦ Explain the potential vicious cycle of perceived breast milk insufficiency
• Highlight and explain the dangers of mixed feeding compared to exclusive breastfeeding
• Discuss the issue of 'bad milk' openly and explain that it does not exist
• Focus on HIV/AIDS and comprehend the participant's fear of stigma
• Encourage already good practices
**Suggested ways to implement the recommendations**
• Use the health personnel and the TBAs, as they are very much trusted in the community
• Improve the training received by the health personnel and TBAs about:
◦ the dangers of mixed feeding, highlighting the importance of explaining this to the mothers
◦ HIV/AIDS and infant feeding
• Increase health education and include the whole community, including fathers, mothers/mothers-in-law and other influential people (i.e. the woman's family) in the education sessions
◦ Use of role models, e.g. mothers who have succeeded with EBF, to educate other mothers
◦ Use many channels and integrate them into other programmes (school teaching, radio, newspapers, etc.)
• Closer follow-up of EBF, for instance by peer-supporters for EBF or by further development and expansion of the exclusive breastfeeding support groups

### Methodological limitations

A possible bias in this study is linked to the recruitment procedure. Nurses, who offered the same women health service, were used to recruit participants to the study. This may have made it difficult for the mother to decline taking part in the study, and furthermore, may have introduced a social desirability bias where the mothers answered what they assumed would be the right thing to do rather than what they actually did.

Another methodological problem was linked to translation. Not being conversant with the local languages, the first author analysed the data from transcripts that had already been translated into English. The translation process may to some extent have altered the original meaning of the text. However, any ambiguity in the English version was checked and compared with the text in the original transcript.

### The content of the health education

Health education in this geographical area should build on the following positive findings of this study: almost every child was breastfed immediately after birth, colostrum was rarely discarded, the use of pre-lacteal feeds appeared low, breastfeeding was almost universal, prolonged breastfeeding was widely practised and the fathers and the grandmothers required the mother to breastfeed. Education should also focus on the five issues listed above. The first is the perception of insufficient milk. This is a well known barrier to EBF [18-21]. When a child is crying, it is assumed that it is not satisfied and is crying for more food. Owing to the physiological need for suckling to maintain milk production, this easily develops into a vicious cycle; the mother gives the child complementary food and breastfeeding becomes less frequent leading to decreased production of breast milk. Thus the idea of insufficient milk is confirmed. None of the participants in this study mentioned that letting the child breastfeed often in order to stimulate the milk production could solve the problem of "insufficient milk". In this area, mixed feeding is the conventional way to feed an infant. In particular, water and other fluids are given at an early age [22], mainly because of the misconception that breast milk contains insufficient water. In this study, the mothers were well informed about many of the benefits of EBF. This is a great improvement from what was documented in a previous Zambian study [23], but the risks of mixed feeding did not seem to be emphasised. The mothers appeared unaware of the increased risk of infectious diseases [24] when giving complementary food and water. In general, complementary food seemed to be introduced far too early, compared to the guidelines (six months) promoted by the health personnel. Consistently with our findings, a previous study from Zambia [23] concluded that the average age at which mixed feeding was introduced was 2–3 months, and this has been verified by other studies [19,25]. The perception of insufficient milk was the main reason for introducing complementary feeds, as other studies have also demonstrated [18-21,23]. The main supplement given, light *mealie meal *porridge, is of poor nutritional value. Its energy content alone is lower than breast milk. It generally gets over-diluted to achieve an appropriate viscosity [25]. Mothers commonly showed great concern that the child should be satisfied, but even though the concept of "balanced diet" was mentioned by some, there seemed to be limited awareness about the nutritional value of the food intake. Inadequate nutritional knowledge may result in unsuitable feeding practices [25]. In order to promote EBF in this area, health education should emphasize the physiology of breastfeeding and the negative effects of early complementary and hence, mixed feeding.

The fear of dying or becoming too sick to be able to breastfeed was another major concern of mothers which motivated the early introduction of complementary food and which reduced the enthusiasm about the idea of exclusive breastfeeding. Owing to the high prevalence of HIV in the area and the risk of dying from other causes, mothers did not want their babies to rely on breastfeeding only. This concern needs to be addressed both as a psychological barrier to EBF and as a knowledge barrier. The challenge is to gain acceptance for the fact that even if weaning is abruptly precipitated the child will be no better off if it had been mixed fed early in life.

HIV/AIDS was only mentioned in discussions about diseases and when not to breastfeed. Among the mothers, there was a great deal of confusion about whether breastfeeding could be continued if the mother was HIV positive. The fathers and the grandmothers said that a HIV positive mother could not breastfeed. The possible protective effect of EBF on the risk of HIV transmission compared to mixed feeding [26-28], was never mentioned by any of the informant groups. The recent study by Coovadia et al makes a strong case for EBF for HIV-positive mothers in low-resource settings [6]. A study in Ndola, Zambia [29], revealed that informing the mothers that HIV could be transmitted through breast milk did not erode good breastfeeding practices. Hence, the issue of HIV and breastfeeding needs to be put on the health education agenda in order to minimise the damage of inappropriate feeding practices that may develop in the wake of the current confusion. However, it is very important that EBF should be promoted as the best infant feeding method for all infants/children. EBF should not be associated with HIV positive status of the mother, as this would undermine the general promotion of EBF as a health enhancing practise for all children.

All the participants were familiar with the expression 'bad milk'. This is a common Central and/or Southern Africa traditional belief and represents a threat to the maintenance of breastfeeding. The belief that the behaviour of the mother can affect the quality of the breast milk and even make it dangerous to the child, needs to be approached and challenged.

The study revealed divergences between the rural and the urban populations. In general, the rural mothers were more likely to be negative towards colostrum and possibly to discard it, to give pre-lacteal feeds, to be sceptical about EBF and thus not practise it, to have a strong belief in 'bad milk' and to practise customary weaning methods. Furthermore, they were less educated about infant feeding, most probably because of inconsistent and more limited health care access. Old people, often the watchdogs of tradition, tended to have more influence, mainly because of the extended family structure still prevalent in the rural village setting. In order to decrease this knowledge gap between the urban and rural populations, there is a need to increase both the quantity and the quality of the health education in rural areas.

The study in general points to the lack of autonomy and decision making power on the part of young mothers. Infant feeding decisions are not left to her alone, but are subject of great concern also to the extended family group. However, the knowledge of EBF is not evenly distributed. Neither the grandmothers nor the fathers were well informed about EBF; many of them had not heard about it. The same people also expressed a lot of scepticism to EBF, some to the extent that they would try to prevent their wife/daughter/daughter-in-law from practising it. The family pressure may put the young mother in the difficult situation having to make decisions based on contradictory advice: the health personnel at the clinic, whom she trusts, giving her one kind of advice, and the family members at home giving her another. In this context, the promotion of EBF needs to target not only the childbearing woman, but also her close kin, including her husband, her mother and her mother-in-law. Furthermore it is important to continue to strengthen women's decision making power through general empowerment strategies including education and income generation.

### Implementation of health education

The question is how the knowledge and trust in EBF can be strengthened in Mazabuka District. Both health personnel and the TBAs interviewed in this study were more in favour of EBF than what has been reported previously from Zambia [23]. Health personnel are highly trusted in the area as they are considered educated and knowledgeable. The mothers actively sought advice from them and clearly expressed their motivation to follow their guidance. This position of authority makes health personnel key resources in the promotion of good infant feeding practices, including EBF. However, scarcity of resources and nursing staff in the rural areas limits the potential of health care professionals. Alternative approaches must be sought. This study shows that in general, TBAs are more knowledgeable and updated about infant feeding than other non-professionals. The possibility of mobilising and training TBAs for infant feeding counselling should be further explored.

The study also revealed some gaps in the health education given by the health personnel. The risks of mixed feeding in general and in terms of HIV transmission in particular were never brought up, not even when talking about the benefits of EBF. For the success of EBF promotion to the general public, it is of vital importance that it does not become to closely linked to HIV. Nevertheless, the general poor knowledge of the mothers on prevention of mother to child transmission of HIV calls for a greater effort to integrate the issue into the regular health education sessions at the antenatal clinic.

Although the mothers were recommended to practise EBF, it was never actively followed up and a mother in doubt was at high risk of returning to the traditional practice of mixed feeding. Postnatal home visits and community peer-supporters for EBF have been shown to be effective in increasing the proportion of mothers who breastfeed exclusively [8-10,30]. Exclusive breastfeeding support groups already exist in this area, although they were not mentioned by any of the mothers in the study. A previous study concluded that the knowledge in the group was superior to that of the health providers [23]. These groups could be expanded and organised so that they can reach out to the community and be used actively to promote EBF.

## Conclusion

The message that EBF is a useful tool for improving child health had reached the health workers and was being passed on to the mothers. However, conventions on infant feeding and expectations from family members in this Zambian community were important barriers, preventing the translation of the message of EBF into practice. This needs to be taken into account in strategies to support optimal breastfeeding. Important questions in this context are related to the lack of decision making power on the part of the mother and the lack of knowledge of EBF on the part of the fathers and the grandmothers who influence infant feeding. How to target these groups in health education is a challenge that demands immediate attention if promotion of EBF is to succeed.

## Competing interests

The authors declare that they have no competing interests.

## Authors' contributions

All authors participated in the design and planning of the study: the field work was conducted by EF and SS, supported by MK-B and CK; the analysis and write-up was carried out mainly by EF, KMM and TT. All authors read and approved the final manuscript.
